# Butane-1,4-diyl bis­(pyridine-4-carboxyl­ate)

**DOI:** 10.1107/S1600536811023646

**Published:** 2011-06-25

**Authors:** J. Muthukumaran, S. Karthikeyan, G. Satheesh, Bala. Manimaran, R. Krishna

**Affiliations:** a Centre for Bioinformatics, Pondicherry University, Puducherry 605 014, India; b Department of Chemistry, Pondicherry University, Puducherry 605 014, India

## Abstract

The mol­ecule of the title compound, C_16_H_16_N_2_O_4_, lies about an inversion centre; the butane chain adopts an extended zigzag conformation. The dihedral angle between the pyridine ring and the adjacent COO group is 3.52 (s14)°.

## Related literature

For a related structure, see: Brito *et al.* (2010 ▶).
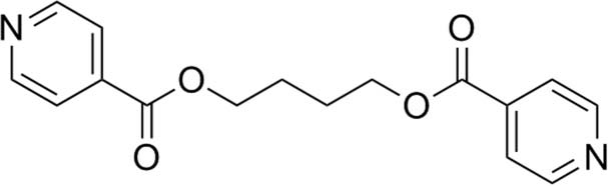


## Experimental

### 

#### Crystal data

C_16_H_16_N_2_O_4_
*M* *_r_* = 300.31
Monoclinic, 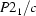

*a* = 7.8519 (5) Å
*b* = 10.5284 (6) Å
*c* = 8.9121 (4) Å
β = 91.770 (5)°
*V* = 736.39 (7) Å^3^
*Z* = 2
Mo *K*α radiation
μ = 0.10 mm^−1^
*T* = 293 K
0.35 × 0.13 × 0.04 mm

#### Data collection

Oxford Diffraction Xcalibur Eos diffractometer
Absorption correction: multi-scan (*CrysAlis PRO*; Oxford Diffraction, 2009 ▶) *T*_min_ = 0.840, *T*_max_ = 1.000
2431 measured reflections
1303 independent reflections
754 reflections with *I* > 2σ(*I*)
*R*_int_ = 0.018

#### Refinement

*R*[*F* ^2^ > 2σ(*F* ^2^)] = 0.039
*wR*(*F* ^2^) = 0.102
*S* = 0.87
1303 reflections
100 parameters
H-atom parameters constrained
Δρ_max_ = 0.18 e Å^−3^
Δρ_min_ = −0.13 e Å^−3^

### 

Data collection: *CrysAlis CCD* (Oxford Diffraction, 2009 ▶); cell refinement: *CrysAlis RED* (Oxford Diffraction, 2009 ▶); data reduction: *CrysAlis RED*; program(s) used to solve structure: *SHELXS97* (Sheldrick, 2008 ▶); program(s) used to refine structure: *SHELXL97* (Sheldrick, 2008 ▶); molecular graphics: *ORTEP-3 for Windows* (Farrugia, 1997 ▶) and *PLATON* (Spek, 2009 ▶); software used to prepare material for publication: *PLATON*.

## Supplementary Material

Crystal structure: contains datablock(s) I, global. DOI: 10.1107/S1600536811023646/ng5184sup1.cif

Structure factors: contains datablock(s) I. DOI: 10.1107/S1600536811023646/ng5184Isup2.hkl

Supplementary material file. DOI: 10.1107/S1600536811023646/ng5184Isup3.cml

Additional supplementary materials:  crystallographic information; 3D view; checkCIF report

## Figures and Tables

**Fig. 1.**
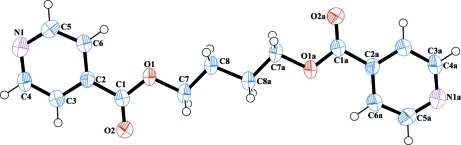
The molecular structure of title compound, showing displacement ellipsoids drawn at the 50% probability level.

**Fig. 2.**
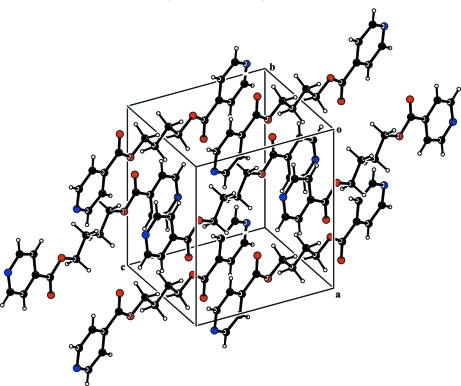
Packing diagram of title compound.

## supplementary crystallographic information

### Comment

Pyridine containing compounds are the new class of anti-HIV molecules, which particularly inhibit RNA dependent DNA polymerase or reverse transcriptase, and hence it acts as non-nucleoside reverse transcriptase inhibitors. They also posses potent anti-bacterial activity. Pyridine containing ruthenium complexes exhibit cytotoxic, anti-cancer, anti-tumor or anti-metastatic activity. Considering the biological importances of the pyridine and its derivatives, a single-crystal of the title compound was prepared for X-ray diffraction studies. The molecular structure of title compound is shown in Fig. 1. The bond distances of pyridyl group in title compound is comparable to those observed in related structure namely propane-1,3-diyl bis(pyridine-4-carboxylate) (Brito *et al.*, 2010). The pyridyl group (N1/C4/C3/C2/C6/C5) adopts a planar conformation (r.m.s. deviation = 0.0019 Å). Cremer & Pople puckering analysis fails, because of its weighted average absolute torsion angle is 0.4°, which is less than 5.0°. The 1,4-butanediyl ester group occupies an equatorial position, which adopt an extended zigzag conformation. Intermolecular π-π stacking interactions is normally found in aromatic compounds. However, in the title compound, the minimal distance between ring centroids is 4.357 (1) Å. Hence, intermolecular π-π stacking interactions are not present in the title compound. The classical hydrogen bonds are not observed. The packing diagram of title compound is shown in Fig. 2.

### Experimental

Isonicotinoyl chloride hydrochloride (639 mg, 3.5 mmol) was taken in a 50 ml round bottom schlenk flask and fitted with a reflux condenser. The system was evacuated and purged with nitrogen. To this, dry dichloromethane 25 ml, 1,4-Butanediol (0.15 ml, 1.7 mmol) and 1 ml of triethylamine were added. The reaction mixture was heated at 40 °C for 5 h. After, the mixture was washed with saturated aqueous sodium bicarbonate solution (20 ml), the organic layer was dried over anhydrous sodium sulfate and filtered. The solvent was evaporated using vacuum and the white product was purified by recrystallization with dichloromethane (Yield: 87%, Melting Point: 140 °C). Single crystals suitable for X-ray diffraction were prepared by slow evaporation of a solution of the title compound in dichloromethane at room temperature. Spectroscopic data of the title compound: IR (KBr): 3046 (*w*), 1728 (*s*), 1560 (*w*), 1476 (*w*), 1286 (*s*), 1127 (*s*), 755 (*m*), cm^-1. 1^H NMR (400 MHz, CDCl_3_): δ 8.77 (d, 4H), 7.83 (d, 4H), 4.43–4.42 (m, 4H), 1.97–1.94 (m, 4H). ^13^C NMR (100 MHz, CDCl_3_): δ 165.2, 150.7, 137.4, 122.9, 65.2, 25.3.

### Refinement

The non-hydrogen atoms were refined anisotropically whereas hydrogen atoms were refined isotropically. The hydrogen atoms were placed in calculated positions (C–H = 0.93–0.97 Å) and included in the refinement in riding-model approximation with *U*_iso_(H) = 1.2Ueq(C).

### Crystal data

**Table:**

C_16_H_16_N_2_O_4_	*F*(000) = 316
*M**_r_* = 300.31	*D*_x_ = 1.354 Mg m^−^^3^
Monoclinic, *P*2_1_/*c*	Mo *K*α radiation, λ = 0.71073 Å
Hall symbol: -P 2ybc	Cell parameters from 852 reflections
*a* = 7.8519 (5) Å	θ = 2.6–28.6°
*b* = 10.5284 (6) Å	µ = 0.10 mm^−^^1^
*c* = 8.9121 (4) Å	*T* = 293 K
β = 91.770 (5)°	Plate, colorless
*V* = 736.39 (7) Å^3^	0.35 × 0.13 × 0.04 mm
*Z* = 2	

### Data collection

**Table:**

Oxford Diffraction Xcalibur Eos diffractometer	1303 independent reflections
Radiation source: fine-focus sealed tube	754 reflections with *I* > 2σ(*I*)
graphite	*R*_int_ = 0.018
Detector resolution: 15.9821 pixels mm^-1^	θ_max_ = 25.0°, θ_min_ = 2.6°
ω scans	*h* = −9→7
Absorption correction: multi-scan (*CrysAlis PRO*; Oxford Diffraction, 2009)	*k* = −12→7
*T*_min_ = 0.840, *T*_max_ = 1.000	*l* = −6→10
2431 measured reflections	

### Refinement

**Table:**

Refinement on *F*^2^	Primary atom site location: structure-invariant direct methods
Least-squares matrix: full	Secondary atom site location: difference Fourier map
*R*[*F*^2^ > 2σ(*F*^2^)] = 0.039	Hydrogen site location: inferred from neighbouring sites
*wR*(*F*^2^) = 0.102	H-atom parameters constrained
*S* = 0.87	*w* = 1/[σ^2^(*F*_o_^2^) + (0.0561*P*)^2^] where *P* = (*F*_o_^2^ + 2*F*_c_^2^)/3
1303 reflections	(Δ/σ)_max_ = 0.001
100 parameters	Δρ_max_ = 0.18 e Å^−^^3^
0 restraints	Δρ_min_ = −0.13 e Å^−^^3^

### Special details

**Table:**

Geometry. All e.s.d.'s (except the e.s.d. in the dihedral angle between two l.s. planes) are estimated using the full covariance matrix. The cell e.s.d.'s are taken into account individually in the estimation of e.s.d.'s in distances, angles and torsion angles; correlations between e.s.d.'s in cell parameters are only used when they are defined by crystal symmetry. An approximate (isotropic) treatment of cell e.s.d.'s is used for estimating e.s.d.'s involving l.s. planes.
Refinement. Refinement of *F*^2^ against ALL reflections. The weighted *R*-factor *wR* and goodness of fit *S* are based on *F*^2^, conventional *R*-factors *R* are based on *F*, with *F* set to zero for negative *F*^2^. The threshold expression of *F*^2^ > σ(*F*^2^) is used only for calculating *R*-factors(gt) *etc*. and is not relevant to the choice of reflections for refinement. *R*-factors based on *F*^2^ are statistically about twice as large as those based on *F*, and *R*- factors based on ALL data will be even larger.

### Fractional atomic coordinates and isotropic or equivalent isotropic displacement parameters (Å^2^)

**Table:**

	*x*	*y*	*z*	*U*_iso_*/*U*_eq_	
O1	0.33092 (14)	0.50761 (11)	0.18817 (12)	0.0510 (4)	
C2	0.21740 (19)	0.51211 (16)	−0.05823 (17)	0.0389 (4)	
C1	0.2787 (2)	0.58307 (18)	0.0775 (2)	0.0466 (5)	
O2	0.2806 (2)	0.69674 (13)	0.08561 (15)	0.0789 (5)	
C3	0.1504 (2)	0.57907 (18)	−0.17878 (19)	0.0504 (5)	
H3	0.1438	0.6672	−0.1753	0.060*	
C6	0.2231 (2)	0.38214 (18)	−0.06986 (19)	0.0529 (5)	
H6	0.2663	0.3327	0.0090	0.063*	
N1	0.0981 (2)	0.39039 (17)	−0.31796 (17)	0.0607 (5)	
C4	0.0936 (2)	0.5144 (2)	−0.3041 (2)	0.0546 (6)	
H4	0.0491	0.5614	−0.3844	0.065*	
C7	0.3923 (2)	0.57013 (18)	0.32514 (19)	0.0539 (6)	
H7A	0.3001	0.6162	0.3705	0.065*	
H7B	0.4819	0.6300	0.3026	0.065*	
C8	0.4589 (2)	0.47077 (18)	0.42959 (18)	0.0465 (5)	
H8A	0.5424	0.4195	0.3792	0.056*	
H8B	0.3663	0.4156	0.4577	0.056*	
C5	0.1632 (3)	0.3269 (2)	−0.2011 (2)	0.0645 (6)	
H5	0.1688	0.2389	−0.2084	0.077*	

### Atomic displacement parameters (Å^2^)

**Table:**

	*U*^11^	*U*^22^	*U*^33^	*U*^12^	*U*^13^	*U*^23^
O1	0.0716 (8)	0.0474 (8)	0.0329 (7)	0.0001 (6)	−0.0175 (6)	−0.0006 (6)
C2	0.0430 (10)	0.0424 (10)	0.0310 (9)	−0.0030 (8)	−0.0023 (8)	−0.0006 (9)
C1	0.0597 (12)	0.0440 (11)	0.0356 (10)	−0.0006 (10)	−0.0063 (9)	0.0026 (10)
O2	0.1401 (14)	0.0422 (9)	0.0522 (9)	−0.0013 (9)	−0.0322 (8)	−0.0016 (7)
C3	0.0659 (13)	0.0436 (11)	0.0410 (11)	−0.0005 (10)	−0.0108 (10)	0.0029 (9)
C6	0.0726 (13)	0.0466 (12)	0.0386 (11)	0.0012 (10)	−0.0110 (10)	0.0022 (10)
N1	0.0755 (12)	0.0600 (12)	0.0454 (10)	−0.0034 (9)	−0.0162 (9)	−0.0042 (9)
C4	0.0683 (14)	0.0590 (13)	0.0353 (10)	0.0028 (11)	−0.0156 (9)	0.0027 (10)
C7	0.0710 (13)	0.0542 (13)	0.0353 (10)	−0.0022 (10)	−0.0185 (10)	−0.0059 (9)
C8	0.0540 (11)	0.0516 (11)	0.0331 (9)	−0.0001 (9)	−0.0111 (8)	−0.0010 (9)
C5	0.0927 (16)	0.0447 (12)	0.0553 (13)	−0.0060 (11)	−0.0134 (12)	−0.0084 (11)

### Geometric parameters (Å, °)

**Table:**

O1—C1	1.322 (2)	N1—C4	1.312 (2)
O1—C7	1.4558 (19)	N1—C5	1.326 (2)
C2—C6	1.373 (2)	C4—H4	0.9300
C2—C3	1.376 (2)	C7—C8	1.485 (2)
C2—C1	1.489 (2)	C7—H7A	0.9700
C1—O2	1.199 (2)	C7—H7B	0.9700
C3—C4	1.371 (2)	C8—C8^i^	1.523 (3)
C3—H3	0.9300	C8—H8A	0.9700
C6—C5	1.376 (2)	C8—H8B	0.9700
C6—H6	0.9300	C5—H5	0.9300
			
C1—O1—C7	116.16 (14)	C3—C4—H4	117.9
C6—C2—C3	117.66 (16)	O1—C7—C8	107.95 (14)
C6—C2—C1	123.43 (17)	O1—C7—H7A	110.1
C3—C2—C1	118.91 (17)	C8—C7—H7A	110.1
O2—C1—O1	123.51 (18)	O1—C7—H7B	110.1
O2—C1—C2	123.57 (18)	C8—C7—H7B	110.1
O1—C1—C2	112.93 (16)	H7A—C7—H7B	108.4
C4—C3—C2	119.25 (17)	C7—C8—C8^i^	111.34 (19)
C4—C3—H3	120.4	C7—C8—H8A	109.4
C2—C3—H3	120.4	C8^i^—C8—H8A	109.4
C2—C6—C5	118.30 (18)	C7—C8—H8B	109.4
C2—C6—H6	120.9	C8^i^—C8—H8B	109.4
C5—C6—H6	120.9	H8A—C8—H8B	108.0
C4—N1—C5	115.97 (17)	N1—C5—C6	124.61 (18)
N1—C4—C3	124.21 (18)	N1—C5—H5	117.7
N1—C4—H4	117.9	C6—C5—H5	117.7
			
C7—O1—C1—O2	0.3 (3)	C3—C2—C6—C5	0.6 (3)
C7—O1—C1—C2	−179.81 (14)	C1—C2—C6—C5	−179.81 (17)
C6—C2—C1—O2	176.70 (18)	C5—N1—C4—C3	−0.3 (3)
C3—C2—C1—O2	−3.7 (3)	C2—C3—C4—N1	0.2 (3)
C6—C2—C1—O1	−3.2 (2)	C1—O1—C7—C8	−175.01 (15)
C3—C2—C1—O1	176.39 (15)	O1—C7—C8—C8^i^	174.67 (17)
C6—C2—C3—C4	−0.3 (3)	C4—N1—C5—C6	0.6 (3)
C1—C2—C3—C4	−179.94 (16)	C2—C6—C5—N1	−0.7 (3)

Symmetry codes: (i) −*x*+1, −*y*+1, −*z*+1.
